# PRDX6 Promotes the Differentiation of Human Mesenchymal Stem (Stromal) Cells to Insulin-Producing Cells

**DOI:** 10.1155/2020/7103053

**Published:** 2020-01-21

**Authors:** Mahmoud M. Gabr, Mahmoud M. Zakaria, Ayman F. Refaie, Sherry M. Khater, Sylvia A. Ashamallah, Sahar A. Rashed, Ali M. Fouad, Amani M. Ismail, Mohamed A. Ghoneim

**Affiliations:** The Urology and Nephrology Center, Mansoura, Egypt

## Abstract

Mesenchymal stem cells (MSCs) can be differentiated in vitro to form insulin-producing cells (IPCs). However, the proportion of induced cells is modest. Extracts from injured pancreata of rodents promoted this differentiation, and three upregulated proteins were identified in these extracts. The aim of this study was to evaluate the potential benefits of adding these proteins to the differentiation medium alone or in combination. Our results indicate that the proportion of IPCs among the protein(s)-supplemented samples was significantly higher than that in the samples with no added proteins. The yield from samples supplemented with PRDX6 alone was 4-fold higher than that from samples without added protein. These findings were also supported by the results of fluorophotometry. Gene expression profiles revealed higher levels among protein-supplemented samples. Significantly higher levels of GGT, SST, Glut-2, and MafB expression were noted among PRDX6-treated samples. There was a stepwise increase in the release of insulin and c-peptide, as a function of increasing glucose concentrations, indicating that the differentiated cells were glucose sensitive and insulin responsive. PRDX6 exerts its beneficial effects as a result of its biological antioxidant properties. Considering its ease of use as a single protein, PRDX6 is now routinely used in our differentiation protocols.

## 1. Introduction

We provided evidence that a modest proportion (3–5%) of mesenchymal (stromal) stem cells obtained from human bone marrow (HBM-MSCs) and from adipose tissue (HAT-MSCs) can be differentiated to form insulin-producing cells (IPCs) [1]. Transplantation of these cells under the renal capsule of chemically induced diabetic nude mice resulted in control of diabetes [2]. We also demonstrated that the transplanted cells undergo further differentiation in vivo. The proportion of IPCs in the harvested kidneys increased to a peak of ~18% 4 weeks after transplantation, without a substantial change thereafter [3]. This finding suggests that this change could be the result of favourable factor(s) in the in vivo micro-environment.

As early as 1999, it was reported that a cytosolic extract from a regenerating pancreas after injury could treat streptozotocin (STZ)-induced diabetes in BALB/c mice [4, 5]. Later, it was observed that an extract from the injured pancreas can also promote the differentiation of rat mesenchymal stem cells into IPCs [6, 7]. In a proteomics-based study, Xie and associates identified 4 proteins that were differentially expressed in extracts from the injured pancreas of Sprague Dawley (SD) rats [8]. Among these 4 proteins, the expression of cofilin-1, nucleoside diphosphate kinase A (NDPKA) and peroxiredoxin-6 (PRDX6) increased. However, the expression of the mitochondrial serine protease HTRA2 decreased. These proteins may have a key role in promoting the differentiation of stem cells into IPCs.

Herein, we report the results of supplementation with these three upregulated proteins, alone or in combination, on the efficiency of HAT-MSC differentiation to IPCs.

## 2. Methods

### 2.1. Recruitment of MSCs

The required approval for this study was obtained from the ethical committee of the University of Mansoura. Liposuction aspirates were obtained from 3 consenting healthy subjects during elective cosmetic surgeries.

### 2.2. Isolation and Expansion of HAT-MSCs

The aspirates were digested by 0.075% collagenase type I (Sigma-Aldrich, St. Louis, USA) for 30 min at 37°C with gentle stirring. The collagenase was inactivated with an equal volume of complete medium (DMEM/10% foetal bovine serum) and centrifuged for 10 min at 300 × g. The cellular pellet was resuspended in DMEM supplemented with 10% foetal bovine serum (FBS) and filtered through a 100 *µ*m mesh filter to remove debris. The resuspended cells were plated at a density of 1 × 10^5^/cm^3^ into 75 cm^2^ culture flasks and incubated at 37°C in a 5% CO_2_ incubator.

Three days later, the nonadherent cells were discarded. The adherent cells were cultured to 80% confluence before passaging with trypsin. The cells were re-cultured in complete DMEM, re-plated at a ratio of 1 : 2 and cultured for another ~8 days to reach 80% confluence. This step was repeated for 3 passages. At this point, the cells were spindle-shaped and displayed a fibroblast-like appearance.

### 2.3. Characterization of the Isolated HAT-MSCs

#### 2.3.1. Phenotyping

MSCs at passage 3 were trypsinized, centrifuged at 300 × g for 8 min, and resuspended in PBS at a concentration of 1 × 10^6^ cells/mL. Aliquots of 100 *µ*L were incubated for 30 min in 20 *µ*L of antibodies specific for CD14/CD45 (FITC) or CD73/CD34 phycoerythrin (PE) or in 5 *µ*L of CD105 (PE) or CD90 (FITC) (Becton-Dickinson, USA) washed with 1 mL of staining buffer (BD-Pharmingen, USA) and resuspended in 500 *µ*L of staining buffer. The labelled cells were analysed using an argon ion laser at a wavelength of 488 nm (FACSCalibur, Becton-Dickinson). A total of ten thousand events were obtained and analysed using Cell Quest software (Becton-Dickinson). Control staining using the appropriate isotype-matched monoclonal antibodies was included.

#### 2.3.2. Multilineage Differentiation Potential

AT-MSCs were induced to differentiate into adipocytes, chondrocytes, and osteocytes using a previously described differentiation protocol [9]. Oil red O was used to stain adipocytes; Alcian blue was used to stain chondrocytes; and alizarin-red was used to stain osteocytes.

### 2.4. Differentiation of HAT-MSCs into IPCs

For immunocytochemistry, cells were cultured in chamber slides (Thermo Scientific, Rochester, NY, USA) at a density of 8 × 10^4^ cells/well. For fluorophotometry, cells were cultured in 24-well plates (Greiner Bio-one, Solingen, Germany) at a density of 7 × 10^4^ cells/well. Differentiation was carried out according to a protocol reported previously by Tayaramma et al. with some modifications [10]. Initially, the cells were cultured in serum-free DMEM supplemented with 1% dimethyl sulfoxide (DMSO) for one day (Sigma-Aldrich). The medium was then replaced with serum-free DMEM containing 100 ng/mL activin-A (R&D systems Inc. Minneapolis, USA), 3 *µ*M CHIR99021 (Sigma-Aldrich) and 100 nM wortmannin (ENZO Life Sciences Inc., NY, USA) for 2 days. The medium was then replaced with serum-free DMEM supplemented with 100 ng/mL activin-A and 3 *µ*M CHIR99021 for 2 more days. Thereafter, the cells were cultured in serum-free DMEM with 55 nM trichostatin-A (TSA, Sigma-Aldrich) was added. Finally, the cells were cultured for an additional 7 days in high-glucose (25 mM) medium containing DMEM:DMEM/F12 at a ratio of 1 : 1. This mixture was supplemented with 10 FBS, 10 nM glucagon-like peptide-1 (GLP-1, Sigma-Aldrich) and a total of 10 ng/mL of the upregulated proteins cofilin-1, NDPKA and PRDX6 (Sigma-Aldrich) as single components or in combination.

### 2.5. Immunolabelling

The utilized primary antibodies included mouse monoclonal anti-human insulin and rabbit polyclonal anti-human c-peptide antibodies (Cell Signaling, Denver, USA). The employed secondary antibodies were Alexa Fluor 488-conjugated anti-mouse IgG (H+L) and Alexa Fluor 555-conjugated anti-rabbit IgG (Cell Signaling). The cells were fixed in 4% paraformaldehyde, permeabilized with chilled 100% methanol for 10 min, blocked with 5% normal goat serum for 60 min at RT and incubated with the primary antibodies overnight at 4°C. The cells were then washed with PBS and incubated with the secondary antibodies for 2 hours.

### 2.6. Immunocytochemistry

Nuclei were stained with DAPI. Negative controls were provided by omitting incubation with the primary antibodies. Confocal images were captured using a Leica TCS-SP8 microscope (Leica Microsystems, Mannheim, Germany). ImageJ software (developed by NIH) was used to determine the proportion of IPCs. To this end, ten fields/well were randomly selected for cell counting, which was carried out by 2 independent histopathologists. The results were expressed as the mean proportion of IPCs among the total number of cells/well.

### 2.7. Quantitation of Cellular Insulin Content by Fluorophotometry

Differentiated cells in medium supplemented with a single protein or a combination of proteins were randomly assigned to at least 2 wells in each of the studied plates. In addition to blanks (medium only), undifferentiated cells, and differentiated cells without an added protein served as negative controls.

Quantitative analysis of the cellular insulin content in each well was carried out by a fluorescence microplate reader (Spark M10, Tecan GmbH, Grödig, Austria). Initially, the plate geometry edit was performed to verify the plate configuration using an empty plate. The following parameters were selected from the supplied Spark Control Magellan Software (Tecan, Austria) to detect fluorescence intensity. The high-energy xenon lamp was set up to flash 30 times. A wavelength of 485 nm was used for excitation, and a wavelength of 535 nm was used for emission in the bottom reading mode with sixteen multiple area reads/well. The mean fluorescent dye intensity of each well was calculated following blank reduction. The results were expressed as the numerical fluorescence intensity and as a colorimetric index.

### 2.8. Gene Expression by Real-Time PCR

Total RNA was extracted from the undifferentiated cells and cells at the end of in vitro differentiation using a Direct-Zol^TM^ RNA miniprep kit (Zymo Research, California, USA). The RNA concentration was measured by a spectrophotometer (Nanodrop 2000, Thermo Fisher Scientific, Massachusetts, USA). Thereafter, three micrograms of total RNA was converted to cDNA using an RT^2^ First Strand Kit (Qiagen Sciences, Germantown, MD, USA). Primers were designed at the website of the Biotechnology Information (NCBI), as shown in Supplemental Table 1. In this study, the expression of the relevant pancreatic endocrine genes was evaluated. Expression was determined for the following genes: the pancreatic endocrine hormones insulin (INS), glucagon (GCG), and somatostatin (SST); the relevant transcription factors pancreatic and duodenal homeobox 1 (PDX1), neurogenin3 (Ngn3), regulatory factor X6 (RFX6), neurogenic differentiation factor 1 (NeuroD1), and V-maf musculoaponeurotic fibrosarcoma oncogene homologue A and B (MafA & MafB); the pancreatic enzyme glucokinase (GCK); the glucose transporter solute carrier family member 2 (GLUT-2); the endocrine precursor marker nestin (NES); and the nuclear hormone receptor superfamily member estrogen-related receptor gamma (ERR*γ*). Glyceraldehyde-3-phosphate dehydrogenase (GAPDH) was included as an internal control and for normalization. Amplifications were performed for each sample using a 20 *µ*L reaction volume consisting of 10 *µ*L of 2X Maxima SYBR Green Master Mix (Thermo Fisher Scientific), 2 *µ*L of primers (5 pmol), 1 *µ*L of cDNA template (100 nmol), and 7 *µ*L of nuclease- free water. The evaluation was carried out in a 96-well plate inserted into a real-time thermal cycler (CFX96 Real-Time System, Bio-Rad, Hercules, CA, USA). The cycling parameters for PCR amplification were programmed as follows: initial denaturation at 95°C for 3 min, followed by 40 cycles of denaturation at 95°C for 15 seconds, annealing at 60°C for 30 seconds and extension at 72°C for 30 seconds. The procedure was performed in triplicate for each sample. A mathematical model introduced by Pfaffl [11] was used for relative quantification of the target genes. In this study, the results were expressed relative to those of undifferentiated MSCs.

### 2.9. In Vitro Insulin and c-peptide Release in Response to Increasing Glucose Concentrations

One million cells were initially incubated for 3 hours in glucose-free Krebs-Ringer bicarbonate buffer (KRB), followed by incubation for 1 hour in 3.0 mL of KRB containing 5.5, 12, or 25 mM glucose. The supernatant was collected at the end of each incubation period. The collected samples were frozen at −70°C until they were assayed using an Elisa kit with a minimum detection limit of 1.76 *µ*IU/mL (DRG Diagnostic, Germany). Finally, the protein content of each sample was determined by the Bradford method using a spectrophotometer (Azzota Corporation, Claymont, DE, USA). The results were expressed as ng/*µ*g protein/hr.

### 2.10. Statistical Analysis

For more than one comparison of continuous unmatched data, the *F* test (ANOVA) was used. The Scheffe test was then employed to determine which comparison or comparison(s) contributed to the overall difference. A *p*-value of <0.05 was considered significant.

## 3. Results

### 3.1. Characteristic Features of HAT-MSCs

The cells adhered to plastic and exhibited a spindle-shaped morphology. Phenotypically, the cells were strongly positive for the MSC surface markers CD73, CD90, and CD105 and were negative for the haematopoietic stem cell surface markers CD14, CD34, and CD45 (Supplemental Table 2). In addition, the cells could be differentiated to form adipocytes, chondrocytes and osteocytes when the appropriate growth factors were used (Supplemental Figure 1).

### 3.2. Immunofluorescence

#### 3.2.1. Confocal Microscopy

**Figure 1:**
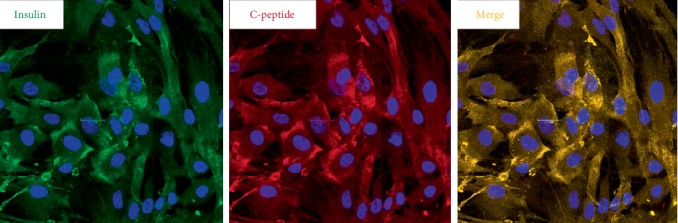
Immunofluorescence of HAT-MSCs following in vitro differentiation. PRDX6 was added to the differentiation medium. The cells exhibited insulin-positive granules (green) and c-peptide (red). Insulin and c-peptide were co-expressed within the same cell.

By immunofluorescence, the differentiated cells were positive for insulin and c-peptide. Insulin and c-peptide were co-expressed within the same cells (Figure 1).

**Figure 2:**
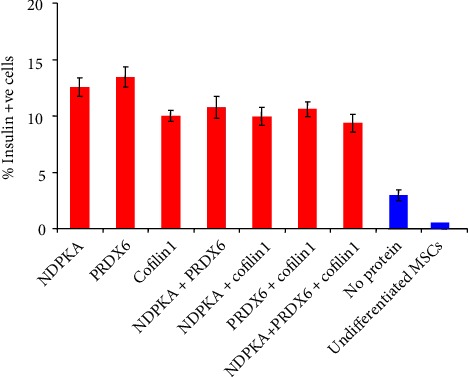
Proportions of IPCs following directed differentiation. The yield of IPCs from all samples supplemented with a protein(s) was significantly higher than that from samples without protein addition. PRDX6-supplemented samples exhibited the highest proportion (13.5%), which represents a 4-fold increase relative to supplemented samples.

The proportions of IPCs among samples in which a protein was added were significantly higher than those among samples without a supplementary protein. The addition of PRDX6 alone increased the yield of IPCs by ~4-fold. However, compared with the other proteins, the addition of PRDX6 provided a marginal advantage that did not reach statistical significance (Figure 2, Supplemental Table 3).

#### 3.2.2. Quantitative Fluorophotometry

**Figure 3:**
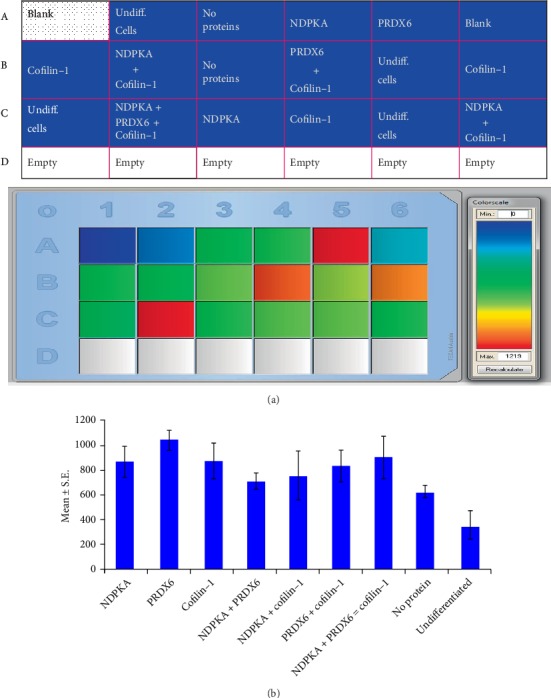
(a) Quantitative immunofluorescence by fluorophotometry. Colorimetric index revealed that the wells containing PRDX6- supplemented differentiation medium had the highest emission. (b) Numerical fluorescence intensity: samples supplemented with a protein(s) exhibited significantly higher values than those without an added protein.

The intensity of the emitted fluorescence as a function of the insulin protein content within the differentiated cells was evaluated as a colorimetric index (Figure 3(a)) and as a numerical fluorescence intensity value (Figure 3(b), Supplemental Table 4). By both methods, PRDX6-supplemented samples exhibited higher readings, but this difference did not reach statistical significance.

### 3.3. Relative Gene Expression by Real-Time PCR

**Figure 4:**
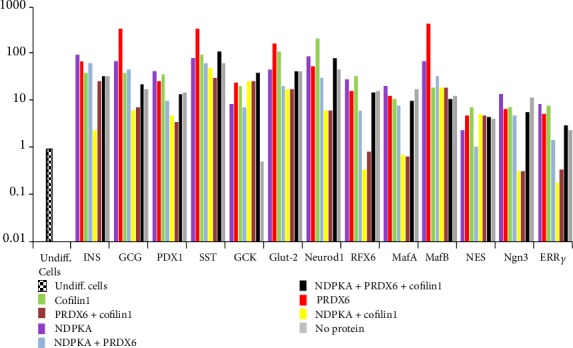
Relative gene expression by real-time PCR. At the end of differentiation, the relevant pancreatic endocrine genes were expressed by all samples. There were significantly higher levels of GCG, SST, Glut-2, and MafB expression among samples supplemented with PRDX6. The expression of ERR*γ* increased when NDPKA, PRDX6 or cofilin-1 was added to the differentiation medium.

At the end of differentiation, the studied endocrine pancreatic genes were expressed among all the different samples (Figure 4). There were significantly higher levels of GCG, SST, Glut-2, and MafB expression in samples supplemented with PRDX6. Notably, expression of the ERR*γ* gene was also increased, particularly when NDPKA, PRDX6, and cofilin-1 were added to the medium (Supplemental Table 5).

### 3.4. Insulin and c-peptide Release (Figure 5)

There was a stepwise increase in the release of insulin and c-peptide as a function of increasing glucose concentrations. At a glucose concentration of 25 mM, this increase was greater among samples supplemented with PRDX6. However, this difference did not reach statistical significance (Supplemental Table 6).

## 4. Discussion

The use of MSCs for cell replacement therapies offers several distinct advantages considering their availability and abundance. MSCs are easy to cultivate and expand and can maintain their multilineage differentiation potential following prolonged culture [9]. They are nonteratogenic, and their use is free of any ethical considerations. While autologous application of MSCs should be very safe, these cells also have potential for use in the allogenic setting since their expression of the HLA-DR antigen is very weak [12], and their immunomodulatory function has been reported by several authors [13–16]. In this study, we used HAT-MSCs, which are available in large quantities from liposuction aspirates. Thus, the trauma associated with the collection of bone marrow samples is avoided. Furthermore, one gram of adipose tissue yields ≃5000 stem cells, whereas the yield from bone marrow is only 100–1000 cells/mL [17].

Initially, several investigators tried to induce HBM-MSCs from rodents to generate IPCs [18–20]. Subsequently, Sun et al. reported successful differentiation of HBM-MSCs from diabetic patients into IPCs in vitro [21]. These early reports were met with scepticism. Some experts argued that the presence of insulin in such cells is the result of absorption and sequestration of insulin from the culture medium [22]. Later, objective evidence confirmed that the presence of insulin in these cells is the result of intrinsic synthesis [2, 23, 24]. To this end, several differentiation protocols were tested. In general, the reported yield of IPCs was modest [25, 26]. In our laboratory, the proportion of induced cells was 3–5% when HBM-MSCs were used [2]. In a comparative study, 3 different protocols were evaluated. The end results were similar [27]. Again, the outcomes were not different when bone marrow or adipose tissue was the source for MSCs [1]. In this study, differentiation was carried out according to a previously reported 2-step protocol [10] which was evaluated in our laboratory [27]. The essential feature of this method is the use of TSA and GLP-1. Evidence showed that TSA can result in the differentiation of bone marrow cells into IPCs in the presence of high glucose concentrations and GLP-1 [10, 27, 28]. The modified protocol entails adding activin-A, CHIR99021 and wortmannin during the initial phase of differentiation. These molecules induce stem cells towards a definitive endoderm and can enhance their differentiation into the pancreatic endodermal lineage [29–31]. Deepa et al. also used a different 2-step protocol to induce differentiation of canine bone marrow mesenchymal stem cells into islet-like cells [32]. This method involved the utilization of *β*-mercaptoethanol and nicotinamide for differentiation. The outstanding advantage of this technique is the very short time required for its completion.

Insights into the mechanisms involved in islet regeneration following a stressful insult can reveal new approaches for the treatment of diabetes or identify factor(s) that promote efficient differentiation of stem cells to form IPCs if cell therapy is considered. Several investigators have reported that an extract from the regenerating pancreas after injury was able to cure STZ- induced diabetes in BALB/c mice [4, 5]. Furthermore, it was observed that such an extract could promote the differentiation of MSCs into IPCs [6, 7]. Using a proteomic-based study, increased expression of 3 proteins (NDPKA, PRDX6, and cofilin-1) from extracts of injured pancreata of SD rats were identified [8].

The objective of this investigation was to explore the potential of these proteins, used as single molecules or in combination, for improving the outcomes of differentiation of HAT-MSCs to form IPCs. The utilized proteins were initially titrated using 5, 10, 20, and 30 ng/mL. The 10 ng/mL dose had the most pronounced effect for induction of differentiation. There was no further increase when higher doses were utilized. Intergroup comparisons were carried out and contrasted with samples devoid of supplemented protein(s). Data from undifferentiated cells served as a negative control. The end points for evaluation included the proportion of induced cells by immunocytochemistry, quantitative fluorescence by fluorophotometry, expression of the relevant pancreatic endocrine genes and insulin and c-peptide released in response to increasing glucose concentrations. The proportion of IPCs among samples supplemented with a protein was significantly higher than that among samples without a protein. In comparison with other proteins, as single molecules or in combination, PRDX6 exhibited an advantage that did not reach statistical significance. Notably, the impact of PRDX6 was better as a single molecule than in any combination. PRDX6 increased the proportion of induced cells by ~4-fold relative to that of samples in which a protein was not added (from 2.94% to 13.46%). The emitted fluorescence as a function of the insulin protein content was higher among samples supplemented with PRDX6. However, differences were only significant when samples not supplemented with a protein were considered. At the end of differentiation, all relevant pancreatic endocrine genes were expressed. Significantly higher levels of GGT, SST, Glut-2, and MafB expression were noted among PRDX6- treated samples. In addition, the expression of the estrogen-related receptor gamma gene (ESRRG) was also increased, particularly among samples supplemented with NDPKA, PRDX6 and cofilin-1. In humans, ESRRG encodes the nuclear receptor ERRY [33]. ERRY has a key role in β-cell metabolic maturation and is required for glucose-stimulated insulin secretion [34, 35]. With increasing glucose concentrations, there was a stepwise increase in insulin and c-peptide release by the differentiated cells. This finding indicates that these differentiated cells became glucose sensitive and insulin responsive. Again, PRDX6-treated samples released higher quantities of insulin and c-peptide, particularly at a glucose concentration of 25 mM.

Peroxiredoxins are proteins that act as antioxidant enzymes and are widely distributed in living organisms. Six members of this family were found in mammals and were classified into 3 subgroups based on the number of conserved cysteine (Cys) residues in the PRDX6 monomer: typical 2-Cys (PRDX1–PRDX4), atypical 2-Cys (PRDX5), and 1-Cys (PRDX6) [36]. PRDX6 is similar to other PRDXs in being capable of reducing various peroxides. In contrast to the 2-Cys PRDXs, PRDX6 uses glutathione rather than thioredoxin as the physiological reductant. PRDX6 is distributed throughout all organs, with especially high concentrations in the lung, liver, kidney and testis [37]. It was also reported that PRDX6 is abundant in *β*-cells [38] and that muscle oxidative stress accelerates the shortening of telomeres, ultimately leading to cellular senescence [39]. PRDX6 can neutralize peroxides, peroxynitrites, and phospholipid hydroperoxides [36]. In addition, PRDX6 has a role in the repair of peroxidized cell membranes due to its ability to reduce peroxidized cell membrane phospholipids [37]. In a previous publication, an increase in the levels of reactive oxygen species (ROS) was noted during the differentiation of HAT-MSCs [1]. This observation can provide an explanation for the observed advantage when PRDX6 was added to the differentiation medium. Pacifici et al. demonstrated that PRDX6 −/− mice spontaneously develop a metabolic defect resembling early-stage T2DM. This condition was characterized by higher glucose levels and reduced insulin secretion in response to glucose [38]. On this basis, the functional activity of PRDX6 against oxidative stress and inflammation may be useful in the development of preventive and novel tools for the treatment of T2DM [39].

## 5. Conclusion

As a result of its antioxidant properties, the addition of PRDX6 substantially improved the yield of IPCs following directed differentiation of HAT-MSCs. Currently, PRDX6 is routinely used to supplement our differentiation medium. It is worth mentioning that the proteins that were identified by Xie et al. [8] and evaluated in this investigation were obtained from extracts of injured pancreata from SD rats. Would the results be different if a pancreatic insult was inflicted in the human setting? To address this question, a proteomic-based study of serum samples from patients undergoing partial pancreatectomy is currently underway.

## Figures and Tables

**Figure 5:**
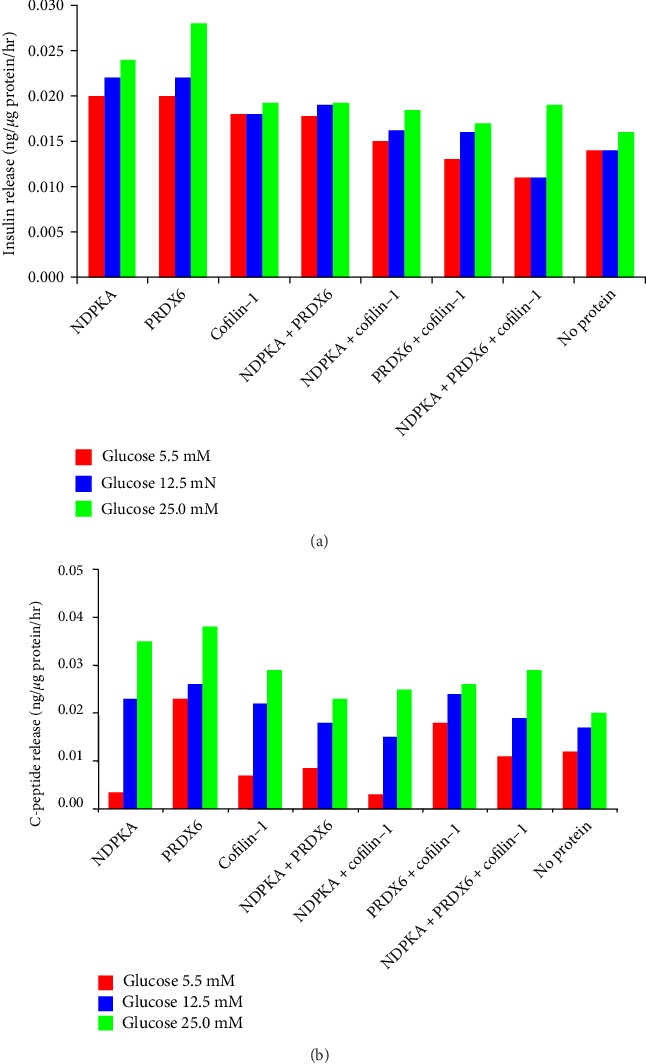
Insulin and c-peptide release. There was a stepwise increase in the release of insulin (a) and c-peptide (b) in response to increasing glucose concentrations. These findings indicate that differentiated IPCs are glucose sensitive and insulin responsive. At a glucose concentration of 25 mM, this increase was greater among samples supplemented with PRDX6. Differences were not statistically significant.

## Data Availability

Data used to support the findings of this study are included within the supplementary information files.

## Supplementary Materials

Supplementary Materials Supplemental Table 1: List of human gene-specific primers for RT-PCR. Supplemental Table 2: Phenotype Characteristics. Supplemental Table 3: Proportion of Insulin-Producing Cells (%). Supplemental Table 4: Quantitative Immunofluorescence: Results from 3 experiments. Supplemental Table 5: Gene Expression of AT-MSCs using the TSA+GLP1 Protocol with Different Added Proteins by Real-Time PCR. Supplemental Table 6: In vitro human insulin and C-peptide release from IPCs derived from HAT-MSCs.
