# Supplementary material for: PRDX6 Promotes the Differentiation of Human Mesenchymal Stem (Stromal) Cells to Insulin-Producing Cells
Source: Biomed Res Int. 2020 Jan 21;2020:7103053. doi: 10.1155/2020/7103053 (PMC6995490; doi:10.1155/2020/7103053)
Supplement: Supplementary Materials — Supplemental Table 1: List of human gene-specific primers for RT-PCR. Supplemental Table 2: Phenotype Characteristics. Supplemental Table 3: Proportion of Insulin-Producing Cells (%). Supplemental Table 4: Quantitative Immunofluorescence: Results from 3 experiments. Supplemental Table 5: Gene Expression of AT-MSCs using the TSA+GLP1 Protocol with Different Added Proteins by Real-Time PCR. Supplemental Table 6: In vitro human insulin and C-peptide release from IPCs derived from HAT-MSCs. [file 7103053.f1.doc]

**Supplemental Table 1**

**List of human gene-specific primers for RT-PCR**

| **Size of PCR**  **product (bp)** | **Reverse primer** | **Forward primer** | **Genes** |  |
| --- | --- | --- | --- | --- |
| **80**  **93**  **96**  **89**  **92**  **86**  **80**  **117**  **83**  **92**  **98**  **90**  **93**  **90** | **TCCTCGCTCTCTTCTCTGCT**  **CGCTTCTTGTCCTCCTCCTT TTGTACAGGTCCCGCTCTTT**  **CCTTGTAGGCGTCTCTCTCG GGTGGCCGGGAGTTATATG GTACAAGCTGTGGTCCGCTA CCCTTGCAAAGCGTCTGAAC**  **cacacttggtctggggatct**  **CACCAACTGCAAAGCTGGAT**  **CACCTTCTCCCACCTTCACC**  **CGGGTCTTGGGTGTGTAGAA**  **cctttcaccagccaagcaat**  **CTCTGCAGCTCAAGCCTCATT**  **TGGTACATGACAAGGTGCGG** | **Endocrine Precursor Marker**  **NES GAGCAGGAGGAGTTGGGTTC**  **Transcription Factors**  **PDX-1 GCTGGCTGTCATGTTGAACT**  **MafA GCACATTCTGGAGAGCGAGA**  **MafB GGAGAATGAGAAGACGCAGC**  **RFX6 CATGGTCGATGCATGGCTTG**  **Ngn3 TCACCAAGATCGAGACGCTG**  **NeuroD1 TACATCTGGGCTCTGTCGGA**  **Estrogen-Related Receptor Gamma**  **ERRγ gacttgactcgccacctctc**  **Glucose Transporter**  **GLUT-2 TTGGGCTGAGGAAGAGACTG**  **Pancreatic Enzyme**  **GCK CACCCCAGAAGGCTCAGAAG**  **Endocrine Hormones**  **INS GCAGCCTTTGTGAACCAACA**  **GCG gagacatgctgaagggacct**  **SST GTCTGAACCCAACCAGACGG**  **Internal Control**  **GAPDH CCACCACACTGAATCTCCCC** | | |

***PDX-1 (****pancreatic and duodenal homeobox 1),* ***MafA*** *(v-maf musculoaponeurotic fibrosarcoma oncogene homologue A),* ***MafB*** *(v-maf musculoaponeurotic fibrosarcoma oncogene homologue B),* ***RFX6*** *(regulatory factor X6),* ***Ngn3*** *(neurogenin 3),* ***NeuroD1*** *(neurogenic differentiation 1),* ***ERRγ*** *(estrogen-related receptor gamma),* ***GLUT-2*** *(glucose transporter member 2),* ***GCK*** *(glucokinase),* ***GCG*** *(glucagon),* ***SST*** *(somatostatin),* ***GAPDH*** *(glyceraldehyde-3-phosphate dehydrogenase),* ***bp*** *(base pair).*

**Supplemental Table 2**

**Phenotype Characteristics**

|  | | **CD45**  **(%)** | | **CD34**  **(%)** | **CD14**  **(%)** | | **CD165**  **(%)** | **CD90**  **(%)** | **CD73**  **(%)** |
| --- | --- | --- | --- | --- | --- | --- | --- | --- | --- |
| 6.09 | 2.1 | | 0.02 | | 99.1 | 97.5 | | 88.4 | **Donor 1** |
| 0.6 | 1.2 | | 0.05 | | 91.5 | 98.1 | | 99.3 |
| 0.37 | 0.58 | | 0.19 | | 94.3 | 99.5 | | 97.4 |
| 0.16 | 0.77 | | 0.03 | | 95.7 | 99.4 | | 98.1 | **Donor 2** |
| 0.06 | 0.06 | | 0.05 | | 96.6 | 91.7 | | 95.4 |
| 0.04 | 0.09 | | 0.59 | | 91.8 | 92.9 | | 90.8 |
| 0.26 | 2.2 | | 0.08 | | 98.2 | 88.5 | | 90.9 | **Donor 3** |
| 0.5 | 0.4 | | 0.05 | | 94.3 | 97.7 | | 94.2 |
| 3.6 | 0.3 | | 1.1 | | 95.2 | 91.6 | | 98.1 |

**Supplemental Table 3**

**Proportion of Insulin-**Producing Cells (%)

| **Sample** | **NDPKA** | **PRDX6** | **Cofilin-1** | **NDPKA+**  **PRDX6** | **NDPKA + Cofilin-1** | **PRDX6 +**  **Cofilin-1** | **NDPKA + PRDX6 +**  **Cofilin-1** | **No Protein** | **Undifferentiated**  **HAT-MSCs** |
| --- | --- | --- | --- | --- | --- | --- | --- | --- | --- |
| **1** | 9.7 | 18.0 | 7.4 | 13.5 | 7.2 | 12.5 |  |  |  |
|  | 9.3 | 15.9 | 11.3 | 15.6 | 15.4 | 14.7 |  | 2.0 | 0.00 |
|  | 9.1 | 16.4 | 9.1 | 6.7 | 5.0 | 9.5 |  | 1.5 | 0.00 |
|  | 10.0 | 12.6 | 8.4 | 9.5 | 9.3 | 14.5 | 11.7 | 4.0 | 0.00 |
| **2** | 11.2 | 10.0 | 12.5 | 8.6 | 10.7 | 10.0 | 10.9 | 5.0 | 0.00 |
|  | 15.9 | 12.5 | 11.9 | 17.5 | 10.7 | 11.8 | 9.0 | 3.4 | 0.00 |
|  | 14.5 | 17.0 | 11.5 | 7.8 | 7.5 | 7.6 | 10.3 | 2.0 | 0.00 |
|  | 12.9 | 16.1 | 11.1 | 7.9 | 7.7 | 8.2 | 7.5 | 4.1 | 0.00 |
| **3** | 12.5 | 9.0 | 10.0 | 9.7 | 12.0 | 9.7 | 6.8 | 1.5 | 0.00 |
|  | 11.8 | 10.0 | 10.3 | 9.2 | 12.1 | 9.0 |  |  |  |
|  | 17.0 | 12.3 | 7.8 | 11.0 | 10.5 | 9.0 |  |  |  |
|  | 16.7 | 11.8 | 9.0 | 12.0 | 11.3 | 10.7 |  |  |  |
| **Mean** | **12.55** | **13.46** | **10.02** | **10.75** | **9.95** | **10.6** | **9.37** | **2.94** | **0.00** |
| **Standard deviation** | **2.88** | **3.08** | **1.68** | **3.31** | **2.78** | **2.32** | **1.94** | **1.35** | **0.00** |
| **Standard error** | **0.833** | **0.889** | **0.485** | **0.96** | **0.80** | **0.67** | **0.79** | **0.478** | **0.00** |

**Supplemental Table 4**

**Quantitative Immunofluorescence: Results**

**From 3 Experiments**

| **Sample** | **1** | **2** | **3** | **Mean** | **S.E.** |
| --- | --- | --- | --- | --- | --- |
| **NDPKA** | 788 | 687 | 1116 | 863.66 | 129.49 |
| **PRDX6** | 929 | 1219 | 977 | 1041.66 | 89.74 |
| **Cofilin-1** | 801 | 647 | 1160 | 869.33 | 151.98 |
| **NDPKA+PRDX6** | 675 | 599 | 832 | 702.00 | 68.60 |
| **NDPKA+Cofilin-1** | 781 | 1087 | 374 | 747.33 | 206.51 |
| **PRDX6+Cofilin-1** | 877 | 1029 | 584 | 830.00 | 130.59 |
| **NDPKA+PRDX6+Cofilin-1** | 680 | 760 | 1254 | 898.00 | 179.49 |
| **No Protein** | 520 | 620 | 705 | 615.00 | 53.46 |
| **Undifferentiated** | 232 | 205 | 578 | 338.33 | 120.08 |

**Supplemental Table 5**

**Gene Expression of AT-MSCs using the TSA+GLP1 Protocol with Different Added Proteins by Real-Time PCR**

|  | | | | | | | | | | | | | |
| --- | --- | --- | --- | --- | --- | --- | --- | --- | --- | --- | --- | --- | --- |
|  | **Undiff. Cells** | **NDPKA** | | | **Mean** | **PRDX6** | | | **Mean** | **Cofilin-1** | | | **Mean** |
| **INS** | **1** | 87.98 | 109.36 | 104.95 | **100.76** | 50.50 | 77.69 | 74.30 | **67.49** | 45.50 | 34.58 | 35.94 | **38.67** |
| **GCG** | **1** | 49.41 | 117.78 | 37.26 | **68.15** | 517.67 | 302.98 | 203.37 | **341.34** | 37.17 | 41.19 | 46.37 | **41.58** |
| **PDX1** | **1** | 24.85 | 76.47 | 30.24 | **43.85** | 27.17 | 31.46 | 23.02 | **27.21** | 67.28 | 22.18 | 20.63 | **36.70** |
| **SST** | **1** | 59.21 | 100.96 | 86.96 | **82.38** | 257.94 | 410.12 | 364.84 | **344.30** | 100.21 | 92.03 | 89.29 | **93.84** |
| **GCK** | **1** | 5.98 | 10.87 | 8.38 | **8.41** | 43.38 | 9.24 | 23.96 | **25.53** | 48.25 | 6.71 | 5.54 | **20.17** |
| **Glut-2** | **1** | 43.25 | 39.56 | 60.86 | **47.89** | 86.68 | 238.79 | 186.84 | **170.77** | 198.67 | 75.24 | 75.52 | **116.47** |
| **Neurod1** | **1** | 105.41 | 56.67 | 113.54 | **91.87** | 69.66 | 27.11 | 69.49 | **55.42** | 337.15 | 150.52 | 143.10 | **210.25** |
| **RFX6** | **1** | 23.32 | 32.78 | 27.36 | **27.82** | 11.48 | 23.62 | 14.25 | **16.45** | 30.78 | 34.58 | 37.26 | **34.21** |
| **MafA** | **1** | 16.65 | 24.69 | 21.49 | **20.94** | 12.60 | 12.03 | 15.50 | **13.38** | 19.11 | 7.40 | 7.34 | **11.29** |
| **MafB** | **1** | 54.67 | 83.80 | 75.47 | **71.31** | 434.79 | 414.77 | 513.76 | **454.44** | 31.97 | 15.00 | 13.15 | **20.04** |
| **NES** | **1** | 1.61 | 3.00 | 2.32 | **2.31** | 4.94 | 4.96 | 4.70 | **4.87** | 11.47 | 5.15 | 5.03 | **7.22** |
| **Ngn3** | **1** | 10.76 | 16.26 | 15.83 | **14.28** | 8.41 | 3.19 | 9.06 | **6.89** | 4.95 | 8.77 | 8.72 | **7.48** |
| **ERRy** | **1** | 7.41 | 10.21 | 7.78 | **8.47** | 4.83 | 4.25 | 6.32 | **5.13** | 9.14 | 8.86 | 6.58 | **8.19** |

|  | | | | | | | | | | | | |
| --- | --- | --- | --- | --- | --- | --- | --- | --- | --- | --- | --- | --- |
|  | **NDPKA + PRDX6** | | | **Mean** | **Cofilin-1 + NDPKA** | | | **Mean** | **PRDX6 + Cofilin-1** | | | **Mean** |
| **INS** | 35.92 | 99.62 | 62.24 | **65.93** | 3.18 | 1.66 | 2.17 | **2.34** | 33.71 | 23.10 | 22.49 | **26.43** |
| **GCG** | 58.63 | 47.14 | 36.11 | **47.29** | 5.84 | 7.16 | 5.10 | **6.04** | 11.36 | 4.44 | 6.02 | **7.27** |
| **PDX1** | 6.87 | 11.93 | 11.17 | **9.99** | 10.31 | 4.02 | 0.79 | **5.04** | 5.06 | 2.58 | 3.27 | **3.64** |
| **SST** | 37.61 | 86.04 | 64.11 | **62.59** | 58.58 | 45.99 | 53.05 | **52.54** | 31.59 | 30.73 | 35.05 | **32.46** |
| **GCK** | 9.23 | 4.67 | 8.47 | **7.46** | 59.62 | 5.31 | 13.68 | **26.20** | 61.72 | 4.43 | 12.24 | **26.13** |
| **Glut-2** | 20.46 | 15.34 | 29.00 | **21.60** | 36.65 | 4.71 | 11.75 | **17.70** | 37.94 | 3.94 | 10.52 | **17.46** |
| **Neurod1** | 38.60 | 29.59 | 23.85 | **30.68** | 12.32 | 3.50 | 3.40 | **6.41** | 12.76 | 2.92 | 3.04 | **6.24** |
| **RFX6** | 5.81 | 6.47 | 5.85 | **6.04** | 0.32 | 0.56 | 0.14 | **0.34** | 1.04 | 1.14 | 0.31 | **0.83** |
| **MafA** | 7.59 | 8.13 | 8.43 | **8.05** | 0.78 | 0.66 | 0.67 | **0.70** | 0.81 | 0.55 | 0.60 | **0.65** |
| **MafB** | 33.29 | 31.50 | 36.47 | **33.75** | 33.95 | 12.57 | 13.53 | **20.02** | 35.15 | 10.50 | 12.11 | **19.25** |
| **NES** | 0.89 | 1.09 | 1.17 | **1.05** | 9.25 | 3.20 | 3.44 | **5.30** | 9.57 | 2.67 | 3.08 | **5.11** |
| **Ngn3** | 4.56 | 4.82 | 5.28 | **4.89** | 0.45 | 0.30 | 0.24 | **0.33** | 0.46 | 0.25 | 0.21 | **0.31** |
| **ERRy** | 0.80 | 1.64 | 2.14 | **1.53** | 0.20 | 0.17 | 0.18 | **0.18** | 0.37 | 0.28 | 0.36 | **0.34** |

|  | | | | | | | | |
| --- | --- | --- | --- | --- | --- | --- | --- | --- |
|  | **NDPKA + PRDX6 + Cofilin-1** | | | **Mean** | **No protein** | | | **Mean** |
| **INS** | 33.54 | 37.22 | 35.29 | **35.35** | 52.71 | 25.00 | 22.36 | **33.36** |
| **GCG** | 21.88 | 21.13 | 22.77 | **21.93** | 14.08 | 25.21 | 15.03 | **18.11** |
| **PDX1** | 13.07 | 15.32 | 14.26 | **14.22** | 12.18 | 20.54 | 12.20 | **14.97** |
| **SST** | 120.43 | 118.76 | 112.70 | **117.30** | 66.26 | 93.12 | 39.62 | **66.33** |
| **GCK** | 100.10 | 5.07 | 10.65 | **38.61** | 0.52 | 0.63 | 0.45 | **0.53** |
| **Glut-2** | 55.90 | 40.43 | 33.68 | **43.34** | 50.47 | 49.58 | 33.11 | **44.39** |
| **Neurod1** | 106.13 | 77.20 | 58.65 | **80.66** | 60.04 | 36.22 | 44.89 | **47.05** |
| **RFX6** | 17.05 | 16.06 | 14.00 | **15.70** | 18.85 | 21.39 | 8.92 | **16.39** |
| **MafA** | 13.98 | 8.53 | 8.95 | **10.49** | 21.98 | 18.35 | 12.02 | **17.45** |
| **MafB** | 17.49 | 8.47 | 8.16 | **11.37** | 17.08 | 12.65 | 9.38 | **13.04** |
| **NES** | 7.24 | 2.81 | 3.16 | **4.41** | 5.12 | 4.45 | 2.98 | **4.18** |
| **Ngn3** | 7.94 | 5.29 | 3.63 | **5.62** | 16.14 | 12.88 | 7.97 | **12.33** |
| **ERRy** | 2.76 | 2.68 | 4.02 | **3.15** | 3.39 | 2.31 | 1.29 | **2.33** |

**Supplemental Table 6**

**In vitro human insulin and C-peptide release from IPCs derived from ِِHAT-MSCs**

| **C-peptide Release**  **(ng/µg protein/hr)** | | | **Insulin Release**  **(ng/µg protein/hr)** | | |  |
| --- | --- | --- | --- | --- | --- | --- |
| **25** | **12** | **5.5** | **25** | **12** | **5.5** | **Glucose Conc.**  **(mM)**  **Protein** |
| 0.035 | 0.023 | 0.0035 | 0.024 | 0.022 | 0.02 | 1. NDPKA |
| 0.038 | 0.026 | 0.023 | 0.028 | 0.022 | 0.02 | 2. PRDX6 |
| 0.029 | 0.022 | 0.007 | 0.0192 | 0.018 | 0.018 | 3. Cofilin-1 |
| 0.023 | 0.018 | 0.0085 | 0.0192 | 0.019 | 0.0178 | 4. NDPKA + PRDX6 |
| 0.025 | 0.015 | 0.003 | 0.0184 | 0.0162 | 0.015 | 5. NDPKA + Cofilin-1 |
| 0.026 | 0.024 | 0.018 | 0.017 | 0.016 | 0.013 | 6. PRDX6 + Cofilin-1 |
| 0.029 | 0.019 | 0.011 | 0.019 | 0.011 | 0.011 | 7. NDPKA + PRDX6 + Cofilin-1 |
| 0.02 | 0.017 | 0.012 | 0.016 | 0.014 | 0.011 | 8. No Protein |

**Supplemental Figure (1)**


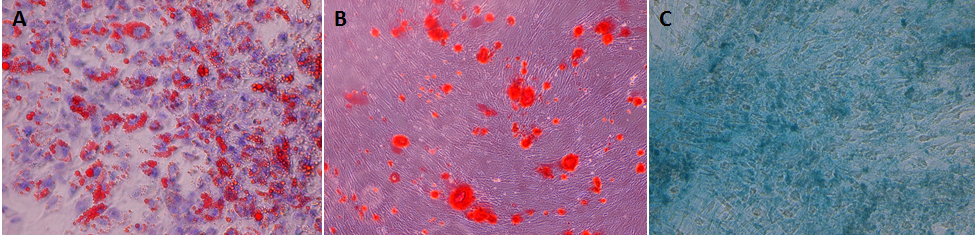


Data used to support the findings of this study are included within the supplementary information files
